# Circulating Tumor DNA in Head and Neck Squamous Cell Carcinoma: Association with Metabolic Tumor Burden Determined with FDG-PET/CT

**DOI:** 10.3390/cancers15153970

**Published:** 2023-08-04

**Authors:** Antti Silvoniemi, Jukka Laine, Katri Aro, Linda Nissi, Leif Bäck, Jukka Schildt, Jussi Hirvonen, Jaana Hagström, Heikki Irjala, Leena-Maija Aaltonen, Marko Seppänen, Heikki Minn

**Affiliations:** 1 Department of Otorhinolaryngology—Head and Neck Surgery, Turku University Hospital, University of Turku, FI-20521 Turku, Finland; 2 Turku PET Centre, University of Turku, FI-20521 Turku, Finland; 3 Department of Pathology, Turku University Hospital, University of Turku, FI-20520 Turku, Finland; 4 Department of Otorhinolaryngology—Head and Neck Surgery, Helsinki University Hospital, University of Helsinki, FI-00029 Helsinki, Finland; 5 Department of Oncology, Turku University Hospital, University of Turku, FI-20521 Turku, Finland; 6 Department of Nuclear Medicine, HUS Diagnostic Center, Helsinki University Hospital, University of Helsinki, FI-00029 Helsinki, Finland; 7 Department of Radiology, Turku University Hospital, University of Turku, FI-20521 Turku, Finland; 8 Department of Radiology, Faculty of Medicine and Health Technology, Tampere University Hospital, Tampere University, FI-33520 Tampere, Finland; 9 Department of Oral Pathology and Radiology, University of Turku, FI-20520 Turku, Finland; 10 Department of Pathology, Helsinki University Hospital, Helsinki University, FI-00290 Helsinki, Finland; 11 Department of Clinical Physiology and Nuclear Medicine, Turku University Hospital, University of Turku, FI-20521 Turku, Finland

**Keywords:** liquid biopsy, ctDNA, circulating tumor DNA, cfDNA, FDG-PET/CT, next-generation sequencing, variant allele frequency, head and neck cancer, metabolic tumor volume, total lesion glycolysis

## Abstract

**Simple Summary:**

The detection of circulating tumor DNA (ctDNA) has gained increasing interest in precision oncology. In head and neck squamous cell carcinoma (HNSCC), a heterogenous mutational landscape contributes to substantial challenges in prognostic and predictive assessment. We report our observations of associations between quantitative parameters in ctDNA and the metabolic tumor burden determined based on FDG-PET/CT. We found that maximum variant allele frequency (VAF) in venous liquid biopsy correlated positively with metabolic tumor burden measured with whole-body total lesion glycolysis (TLG). The prognostic significance of this PET parameter has been documented repeatedly in previous meta-analyses in HNSCC. Our findings indicate that a complex mutational landscape contributes to this metabolic burden. A combination of ctDNA detection and FDG-PET/CT may provide added value for the prognostic and predictive evaluation of HNSCC in the setting of initial diagnosis and follow-up after definitive therapy.

**Abstract:**

Background: The detection of circulating tumor DNA (ctDNA) with next-generation sequencing (NGS) in venous blood is a promising tool for the genomic profiling of head and neck squamous cell carcinoma (HNSCC). The association between ctDNA findings and metabolic tumor burden detected with FDG-PET/CT imaging is of particular interest for developing prognostic and predictive algorithms in HNSCC. Methods: Twenty-six prospectively enrolled HNSCC patients were eligible for further analysis. All patients underwent tumor tissue and venous liquid biopsy sampling and FDG-PET/CT before definitive oncologic treatment. An NGS-based commercial panel was used for a genomic analysis of the samples. Results: Maximum variant allele frequency (VAF) in blood correlated positively with whole-body (WB) metabolic tumor volume (MTV) and total lesion glycolysis (TLG) (r = 0.510, *p* = 0.008 and r = 0.584, *p* = 0.002, respectively). A positive liquid biopsy was associated with high WB-TLG using VAF ≥ 1.00% or ≥5.00% as a cut-off value (*p* = 0.006 or *p* = 0.003, respectively). Additionally, ctDNA detection was associated with WB-TLG when only concordant variants detected in both ctDNA and tissue samples were considered. Conclusions: A high metabolic tumor burden based on FDG imaging is associated with a positive liquid biopsy and high maximum VAF. Our findings suggest a complementary role of metabolic and genomic signatures in the pre-treatment evaluation of HNSCC.

## 1. Introduction

The genomic profiling of head and neck squamous cell carcinoma (HNSCC) has markedly progressed due to modern methods, such as next-generation sequencing (NGS). Liquid biopsy (LB) is a minimally invasive and easily repeatable procedure for detecting biomarkers in body fluids using different technologies such as circulating cell-free DNA (cfDNA), circulating tumor cells, microRNAs, and extracellular vesicles. The detection of circulating tumor DNA (ctDNA) from a venous blood sample provides a picture of the mutational status of malignant disease and holds promise in HNSCC as a favorable method to provide prognostic and predictive information [1,2].

The apoptosis and necrosis of tumor cells are assumed to contribute mainly to the shedding of ctDNA from the neoplastic tissue [3]. Thus, it is reasonable that assaying ctDNA in advanced cancers is a powerful method for detecting the progression of disease [4]. In addition to specific mutations that are feasible for predictive assessment or molecular targeting, some general parameters with prognostic features have been recognized. Of these, a high maximum variant allele frequency (VAF) describes the number of mutated molecules over the total number of wild-type molecules at a specific location in the genome in ctDNA. The maximum VAF has been observed to be associated with poor survival in several cancers [5,6,7]. Additionally, a high number of somatic variants in ctDNA reflects the increased complexity of a tumor and has been shown to confer adverse prognosis [5,8]. Some of these pilot studies have included patients with HNSCC, but disease-specific data on these molecular parameters are limited.

The mutational status detected in tissue sample DNA (tDNA) and ctDNA commonly differ for two main reasons. First, technical issues contribute to the lower sensitivity of ctDNA detection in cases that consider ctDNA as a surrogate marker of primary tumor mutational status. Second, tissue biopsy is typically a snapshot from a single region of a heterogeneous tumor, whereas ctDNA is deemed to reflect the total disease burden where additional clones from metastatic lesions may dominate [9]. Consequently, ctDNA and tDNA should be considered distinct entities providing complementary information. The interpretation of ctDNA findings remains under scrutiny, and supporting information might be needed from other tumor-profiling methods to advance ctDNA’s role in clinical decision-making [10].

FDG-PET/CT imaging is a routinely available imaging modality to investigate metabolic tumor profiles in several cancers, including HNSCC. Metabolic tumor volume (MTV) and total lesion glycolysis (TLG) are quantitative parameters of oncologic FDG-PET/CT, reflecting a metabolic tumor burden. In HNSCC, meta-analyses have indicated the prognostic value of MTV and TLG, where both parameters are associated with inferior disease-free and overall survival [11,12]. A combination of metabolic imaging and cfDNA detection has been shown to provide added value in the prognostic assessment of colorectal cancer [13]. Among solid cancers other than HNSCC, existing links have been observed between quantitative FDG-PET/CT and cfDNA or ctDNA parameters, at least in non-small-cell lung cancer [14,15,16,17], melanoma [18], and colorectal cancer [13]. However, corresponding data in HNSCC are so far limited [19].

Our prospective study examined the association between mutational status on ctDNA/tDNA and the metabolic tumor burden observed in FDG-PET/CT imaging in treatment-naïve HNSCC patients. We aimed to explore the typically diverse mutational and metabolic landscapes of HNSCC [20] to advance the development of optimal prognostic and predictive algorithms for disease profiling.

## 2. Materials and Methods

### 2.1. Study Patients

This prospective study was conducted at Turku University Hospital and Helsinki University Hospital, Finland, between 2019 and 2022. Patients over 18 years of age with newly diagnosed stage III–IV HNSCC, other than cutaneous, sinonasal, and salivary gland carcinoma, and with a WHO performance status of 0–2 were eligible for the study. Patients with oropharyngeal p16-positive T3 SCC with or without neck metastases (stage II) were also eligible. Written informed consent was a prerequisite for participation. Exclusion criteria were a history of former head and neck cancer or other cancer within the last five years, labile diabetes mellitus, and the inability to understand the meaning of the study. The study (NCT03926468) was conducted in accordance with the latest version of the Helsinki Declaration. The study protocol was approved by the Ethics Committee of the Hospital District of Southwest Finland. Turku and Helsinki University Hospitals granted institutional research permissions.

### 2.2. Genomic Studies and Assessment of Genomic Data

Before the onset of oncologic treatment, NGS-based genomic studies were performed for ctDNA and tDNA analyses. Venous blood (2 × 8.5 mL) was obtained and collected in StreckTM Cell-Free DNA tubes, mixed gently for ten minutes, and sent for analysis to Foundation Medicine^®^ Laboratories (Cambridge, MA, USA, or Penzberg, Germany) attached with a clinical requisition form which did not include any information about an ongoing clinical trial. At the beginning of the study, the ctDNA panel (FoundationOne^®^ Liquid) consisted of a completed exonic sequence of 35 genes, introns of 7 genes, and selected exons of an additional 35 genes [21]. The panel was updated during the study, and the updated version (FoundationOne^®^ Liquid CDx) covered 324 genes, including 309 genes with complete exonic coverage [22]. For consistency, only the gene variants covered in both versions of the panel were considered in the study analysis.

A tissue genomic analysis was performed using an NGS-based FoundationOne^®^ CDx test assay detecting the same genes as in the updated liquid biopsy test panel, FoundationOne^®^ Liquid CDx [23]. A histopathologic tumor sample for tDNA analysis was obtained when the diagnostic biopsy was performed. Representative paraffin-embedded tumor tissue was chosen by the study pathologist (J.L. and J.H. (Jaana Hagström)), and ten 5 µm thin sections were sent for analysis to Foundation Medicine^®^ Laboratories (Cambridge, MA, USA, or Penzberg, Germany). All three study procedures (venous sample, tumor sample, and PET/CT imaging) were completed before the first-line treatment.

The study investigators evaluated, in detail, the clinically relevant genomic alterations presented in the Foundation Medicine Reports. Patients were divided into liquid biopsy-positive (LB-+ve) and liquid biopsy-negative (LB-−ve) groups according to the overall finding and the finding of a variant with an allele frequency of ≥1.00% and ≥5.00%, respectively. The maximum VAF in ctDNA was recorded for each patient. Moreover, a variant was considered concordant when a similar variant was observed in the patient’s ctDNA and tissue sample. Then, the previously mentioned grouping procedures were performed again regarding only the concordant variants. Somatic and germline variants are not explicitly separated from each other in the Foundation Medicine Reports. Therefore, the findings were analyzed with and without possible germline variants to relate VAF to maximum somatic allele frequency (MSAF), which refers to genomic alterations associated with a confirmed tumor.

### 2.3. FDG-PET/CT Imaging and Image Analysis

All patients underwent FDG-PET/CT imaging according to institutional standard procedures after fasting for 6–8 h. FDG was administered intravenously as a bolus (mean ± SD dose 284 ± 70 MBq), and PET image acquisition started ~50 min after injection and covered the area between the eyebrows and the kidneys. At Turku University Hospital, Discovery MI (Scanner A) and Discovery VCT PET/CT (Scanner C) (General Electric Healthcare, Milwaukee, WI, USA) were used, and at Helsinki Discovery, MI (Scanner B) and Siemens Biograph 64 (Scanner D) (Siemens Healthineers, Malvern, PA, USA) were used. The sinogram data were corrected for dead time, decay, and photon attenuation and were reconstructed in a 256 × 256 matrix.

Image analyses were performed using the General Electric AW Server 3.2 and Siemens Syngo.via software by two experienced nuclear medicine physicians (M.S. and J.S.) blinded to the patient genomic data. The maximum and mean standardized uptake values (SUVmax and SUVmean) were determined for primary tumors and metastases and solely for primary tumors, respectively. The total metabolic tumor volume (MTV) was determined using 50% of SUVmax as a threshold, and total lesion glycolysis (TLG) was calculated as a product of SUVmean and MTV [24].

### 2.4. Statistical Analysis

Descriptive statistics of means and standard deviations were calculated for continuous variables and counts and percentages for categorical variables. Medians and interquartile ranges (IQRs) are reported for continuous variables with skewed distributions. The comparisons of PET uptake parameters between LB-+ve and LB-−ve groups were assessed using a Mann–Whitney U-test. The Spearman’s rank correlation test was used to investigate the association between maximum VAF and PET parameters. *p* < 0.05 was used as a level of significance (two-tailed). The statistical analyses were performed using IBM SPSS Statistics software version 28.0 (IBM, Chicago, IL, USA).

## 3. Results

### 3.1. Study Population and Sampling

Overall, 34 patients were enrolled from university hospitals in Turku (*n* = 22) and Helsinki (*n* = 12). One patient was excluded because of a tonsillar primary tumor excision before PET/CT imaging, one patient because of a missing primary tumor tissue sample, and six patients because of a failure to receive complete reports from NGS analyses, leaving 26 patients (19 male, 7 female) eligible for further analysis. The average age of the patients at diagnosis was 66.5 years (range 50–86). Table 1 presents the tumor characteristics. The time between LB and FDG-PET/CT imaging was 6.2 ± 5.7 days (range 0–21), and the time between LB and tissue sampling was 25.3 ± 14.7 days (range 2–51). The time for all three study procedures was 27.4 ± 13.1 days (range 6–51; see Supplementary Figure S1).

### 3.2. Genomic Findings in Liquid Biopsies and Tumor Samples

Of the 26 patients, a positive ctDNA detection in blood covering all recovered variants was observed in all but four, translating to a positive LB in 22/26 (85%) of cases. With thresholds of VAF ≥ 1.00% and VAF ≥ 5.00%, the ratios of the positive LB were 17/26 (65%) and 9 (35%), respectively. All 17 of those who had a variant with an allele frequency of ≥1.00% had at least one concordant variant in their ctDNA and tDNA samples. In the whole study population, 56 variants were found in the LB (Supplementary Table S1) in 22/26 (85%) of the patients, and 35 concordant variants were found in 21/26 (81%).

In three patients, a possible germline mutation was observed. These putative germline variants were of the BRCA2, CHEK2, and BRCA1 genes, and their reported allele frequencies in the LB were 48.40%, 50.80%, and 48.40%, respectively. These three patients had at least one additional somatic mutation detected in their ctDNA.

### 3.3. Associations between Genomic Alterations and FDG Uptake in PET/CT Images

**Figure 1:**
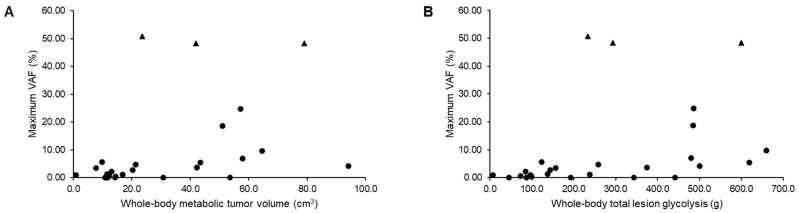
The correlations between maximum variant allele frequency (VAF) and whole-body metabolic tumor volume (MTV, r = 0.510 *p* = 0.008, (**A**)) and maximum VAF and whole-body total lesion glycolysis (TLG, r = 0.584 *p* = 0.002, (**B**)). The triangles refer to the three patients with possible germline mutations, and the others are labeled with circles.

The maximum VAF in ctDNA correlated with the whole-body (WB) MTV (r = 0.510, *p* = 0.008) and WB-TLG (r = 0.584, *p* = 0.002) (Figure 1). The correlations remained significant when the three possible germline mutations were excluded from the analysis (r = 0.411, *p* = 0.037 and r = 0.549, *p* = 0.004, respectively; see Supplementary Figure S2). These correlations were also observed when data from the two main PET/CT vendors were analyzed separately (Supplementary Figure S3). Two patients had higher than median WB-MTV and WB-TLG, despite a negative ctDNA in their LB.

Using VAF ≥ 1.00% as a cut-off value, WB-TLG was higher among the LB-+ve patients than the LB-−ve patients. The similar difference was even larger between groups using a cut-off value VAF ≥ 5.00% (Table 2). WB-MTV was higher in the LB-+ve than the LB-−ve patients with VAF ≥ 1.00% and VAF ≥ 5.00% cut-off values (*p* = 0.045 and *p* = 0.011, respectively). However, these differences in WB-MTV were not significant when the possible germline mutations were excluded from the analysis (*p* = 0.097 and *p* = 0.072, respectively, see Supplementary Table S2). Of the two non-volumetric PET-based indices, the SUVmean of the primary tumor was higher in the LB-+ve than in the LB-−ve patients based on a cut-off value VAF ≥ 1.00% (Table 2). Excluding the abovementioned findings, no associations were observed between PET uptake parameters and the positivity/negativity of VAF ≥ 1.00% or VAF ≥ 5.00% in the LB. The overall ctDNA detection positivity/negativity, regardless of VAF, was not associated with any of the four PET uptake parameters (Table 2).

Apart from the previously mentioned exception (WB-MTV), all results from the analysis, including or excluding the three possible germline mutations, remained comparable (Supplementary Table S2).

Supplementary Figures S4 and S5 and Tables S3 and S4 present the corresponding assessments between PET parameters and genomic alterations, including only the concordant variants. The observed associations were in line with those, including all variants, except that WB-MTV was not in association with LB positivity (VAF ≥ 1.00%), *p* = 0.077.

## 4. Discussion

This study was conducted to explore associations between genomic and metabolic profiles in treatment-naïve HNSCC. We found a clear connection between ctDNA detection and tumor burden measured with metabolic imaging. Maximum VAF was observed to correlate with WB-MTV and WB-TLG in FDG-PET/CT images. In addition, a higher metabolic tumor burden, expressed as WB-TLG, was associated with liquid biopsy positivity using VAF ≥ 1.00% or VAF ≥ 5.00% as a cut-off value. It is important to establish an association between metabolic and genomic profiles in HNSCC to develop prognostic and predictive algorithms for precision medicine. Moreover, more detailed knowledge about the associations of these features might help predict the sensitivity of ctDNA detection on an individual patient level.

The current study population represents typical patients with HNSCC at two Finnish academic hospitals. The study protocol was designed to focus on patients with locally advanced disease due to previous expectations of better performance of ctDNA detection in this patient group than patients with localized disease [4]. The strength of this prospective study was that it allowed a shorter time between ctDNA sampling and PET/CT imaging compared to some previous evaluations [15,18,25]. Therefore, we believe that disease status could be evaluated in a fully comparable manner using two independent methods representing the genomic and functional characteristics of a rapidly progressing cancer.

The metabolic tumor burden determined with FDG-PET/CT reflects tumor growth potential rather than mere geographically defined disease volume. Although they are not in widespread clinical use, MTV and TLG have been observed to hold strong prognostic significance in HNSCC. TLG might be considered the most attractive prognostic index, since it is sensitive to heterogeneous metabolic activity in the tumor region. In addition, the sum of TLG over all lesions has been considered the optimal PET-based estimate for total tumor burden [26]. The common clinical use of SUVmax is mainly based on the easy measurement of this parameter and low observer-dependent variability. However, the prognostic significance of SUVmax is lower than that of MTV and TLG [12,27,28]. In our study, the link between WB-TLG and ctDNA detection was the strongest among all PET-based metabolic parameters. Although a similar relationship seemed to exist between WB-MTV and ctDNA detection, their association was not consistently significant.

We comprehensively assessed the associations between genomic and metabolic parameters in this study. Because of the limited number of patients, we did not attempt to analyze individual mutational changes observed in the LB but concentrated on the magnitude of VAFs as a proxy of genomic pressure inflicted by cancer. In addition to the studies on the correlation between maximum VAF and PET parameters, we explored differences in PET parameters between the LB-+ve and LB-−ve groups divided by dichotomic VAF variables. A cut-off value of VAF ≥ 1.00% was chosen to explore the variants with high expectations to represent the true positive findings [29]. With the current short experience in ctDNA detection in HNSCC, it is highlighted that a remarkable proportion of variants with VAF < 1.00% might also have relevance. However, a high VAF is assumed to be associated with a high likelihood of true positivity [29,30]. Similarly, the challenge of distinguishing true-positive from false-positive findings in ctDNA samples prompted us to perform an analysis limited to concordant variants. Moreover, discordant variants might have a different prognostic impact than concordant variants [9].

The current study’s findings generally align with previous studies comparing the venous blood cfDNA and FDG-PET/CT in several other cancers. A metabolic tumor burden has been observed to correlate with maximum VAF in NSCLC [14], with ctDNA levels in melanoma [18], and with plasma cfDNA concentration in colorectal cancer [13]. On the other hand, some studies have shown a link between WB-SUVmax and ctDNA detection [15,16,25], which was not observed in our study, even in the two subgroups imaged with different PET/CT scanners (Scanners A and B). The diverse mutational landscape and clinical presentation of HNSCC might contribute to these discrepant observations. However, we consider WB-MTV and WB-TLG to be more truthful representations of metabolic tumor burden than WB-SUVmax, which is simply the highest radioactivity of all lesions measured within a volume of a few cubic millimeters. Indeed, our results agree with the previous experiences of the association between ctDNA quantity and tumor burden [31,32]. In HNSCC, the likelihood of a positive liquid biopsy is higher in metastatic than recurrent locoregional disease [31].

Interestingly, two patients in our study had negative ctDNA detection with WB-MTV and WB-TLG that were higher than median. Recently, Woff et al. recognized a minor but distinctive group of colorectal cancer patients with low cfDNA but high MTV. These patients had intermediate prognoses compared to those with high cfDNA/high WB-MTV and low cfDNA/low WB-MTV [13]. Considering Woff et al.’s study and ours, we underscore the individual impact and complementary role of liquid biopsy and metabolic imaging in prognostic evaluations, despite the strong mutual associations observed in most patients.

Three patients in our study had a genomic alteration with a very high VAF that has been previously linked to a germline mutation in the ClinVar database [33]. Unfortunately, we did not perform comprehensive germline testing to distinguish germline mutations from somatic mutations, which would be essential for proving the origin of reproductive cells [34]. Hence, we presented the results, including and excluding the possible germline variants in our analysis. Following this division, we showed that the findings are consistent despite the impact of possible germline variants or discordant variants. The most important exception was the lack of association between WB-MTV and VAF ≥ 1.00% or VAF ≥ 5.00% in the case of omitting the germline variants and the lack of association between WB-MTV and VAF ≥ 1.00% in the case of omitting the discordant variants. When we included only concordant variants and disregarded the possible germline mutations, a significant association between WB-MTV and maximum VAF was maintained. Nonetheless, we consider the association between WB-TLG and ctDNA to be the most robust finding when all comparisons between molecular and imaging metrics are acknowledged.

This study has some limitations. A comprehensive molecular characterization of tumors from a diagnostic biopsy may be limited, although the study’s pathologists carefully selected the slices for tDNA analysis. The number of patients was relatively small and included several subsites in the head and neck area. Therefore, further validation of the results is essential in a larger population. Due to several logistic challenges during the COVID-19 pandemic, we could not schedule PET/CT studies for a single scanner in this prospective study, affecting the consistency of the imaging data. SUVmax and SUVmean are especially susceptible to some variability between different scanners [35], whereas MTV, determined with a predefined percentage of SUVmax, is not as sensitive to interscanner variability [17]. Turku PET Centre and Helsinki University Hospital are members of a European accreditation program [35]. Several procedures have been performed to harmonize PET imaging practice between institutions in this program across several European countries [36]. Furthermore, we performed scanner-specific subgroup analyses for the results of this study and found that they were in line with the analysis of the whole study population.

Some previous studies have even suggested that the metabolic signature in FDG-PET/CT might provide a surrogate for the genomic signature, which is more easily available than the genomic profile from tissue or liquid biopsy [25,37]. Instead of these visions, our objective for this study was different, as we recognize the complexity of HNSCC as a heterogeneous disease. Although the detection of actionable genomic alterations is not yet included in standard therapeutic algorithms, the outlook for biomarker-driven molecular therapy in HNSCC is very encouraging [38]. FDG-PET/CT imaging may assist in the selection of patients likely to have a positive LB as a first step in including them in molecularly targeted therapies. Indeed, we expect that combining ctDNA detection and FDG-PET/CT may provide added value for prognostic and predictive evaluation in the setting of the initial diagnosis and follow-up after definitive therapy. Likewise, our observations may help assess the diagnostic and predictive performance of ctDNA detection at an individual patient level.

## 5. Conclusions

We found an association between high metabolic tumor burden and the detection of somatic variants in ctDNA in patients with locally advanced treatment-naïve HNSCC. A high maximum VAF was associated with a high metabolic tumor burden measured with FDG-PET/CT. A few divergent cases in this study underline the complementary roles of liquid biopsy and metabolic imaging when developing prognostic and predictive algorithms for characterizing HNSCC in the era of precision oncology.

## Figures and Tables

**Table 1:** Tumor characteristics.

		Total (*n* = 26)
Tumor site	Oropharynx	15
	Hypopharynx	5
	Larynx	1
	Oral cavity	5
Stage	II ^a^	1
	III	8
	IV	17
p16	Positive	7
	Negative	18
	Not detected	1

^a^ The only patient with Stage II disease had a cT3N2M0 p16-positive oropharyngeal cancer.

**Table 2:** Median FDG-PET uptake parameters in distinct groups according to liquid biopsy (LB) findings.

	All Patients (*n* = 26)	Any Variant in LB			VAF ≥ 1.00% in LB			VAF ≥ 5.00% in LB		
	Median (IQR)	Positive (*n* = 22)	Negative (*n* = 4)	*p*-Value	Positive (*n* = 17)	Negative (*n* = 9)	*p*-Value	Positive (*n* = 9)	Negative (*n* = 17)	*p*-Value
Whole-body										
SUVmax	16.1 (13.3–22.6)	17.2	15.0	0.471	19.9	14.6	0.220	19.9	14.6	0.220
SUVmean	8.4 (6.5–12.0)	8.5	7.2	0.252	8.5	8.0	0.396	9.5	8.0	0.241
MTV	20.9 (11.9–51.7)	20.9	22.5	0.864	42.0	12.3	0.045	51.1	14.3	0.011
TLG	235.8 (98.5–480.9)	235.8	215.1	0.471	292.5	96.9	0.006	484.6	142.7	0.003
Primary tumor										
SUVmax	13.6 (12.2–19.4)	13.6	14.0	0.811	13.9	12.7	0.339	13.9	13.2	0.711
SUVmean	8.4 (6.7–11.7)	8.6	6.3	0.081	8.6	6.5	0.025	8.6	7.5	0.164
MTV	10.5 (5.4–22.1)	9.1	22.5	0.389	7.8	11.7	0.833	11.8	10.4	0.711
TLG	103.6 (38.8–196.1)	103.6	147.5	0.607	110.0	86.5	0.560	110.0	100.5	0.634

VAF, variant allele frequency; SUV, standardized uptake value; MTV, metabolic tumor volume; TLG, total lesion glycolysis.

## Data Availability

The data presented in this study are available on request from the corresponding author.

## Supplementary Materials

The following supporting information can be downloaded at https://www.mdpi.com/article/10.3390/cancers15153970/s1. Figure S1: Time between liquid biopsy and PET/CT imaging, liquid biopsy and tissue sampling and the time for all three study procedures; Table S1: List of detected variants and their variant allele frequencies (VAF) in ctDNA samples; Figure S2: The correlations between maximum VAF and whole-body metabolic tumor volume, and maximum VAF and whole-body total lesion glycolysis, excluding the possible germline mutations; Figure S3: The correlations between maximum VAF and whole-body metabolic tumor volume, and maximum VAF and whole-body total lesion glycolysis, according to different PET/CT scanners; Table S2: Median FDG-PET uptake parameters in distinct groups according to liquid biopsy findings excluding the possible germline mutations; Figure S4: The correlations between the maximum VAF of concordant variants and whole-body metabolic tumor volume, and maximum VAF of concordant variants and whole-body total lesion glycolysis; Table S3: Median FDG-PET uptake parameters in distinct groups according to liquid biopsy findings regarding only concordant variants; Figure S5: The correlations between maximum VAF of concordant variants and whole-body metabolic tumor volume, and maximum VAF of concordant variants and whole-body total lesion glycolysis, excluding the three possible germline mutations; Table S4: Median FDG-PET uptake parameters in distinct groups according to liquid biopsy findings regarding only concordant variants and excluding the possible germline mutations.
