# Supplementary material for: Circulating Tumor DNA in Head and Neck Squamous Cell Carcinoma: Association with Metabolic Tumor Burden Determined with FDG-PET/CT
Source: Cancers (Basel). 2023 Aug 4;15(15):3970. doi: 10.3390/cancers15153970 (PMC10416934; doi:10.3390/cancers15153970)
Supplement: Supplementary file 1 [file cancers-15-03970-s001.zip › cancers-2472979-supplementary.pdf]

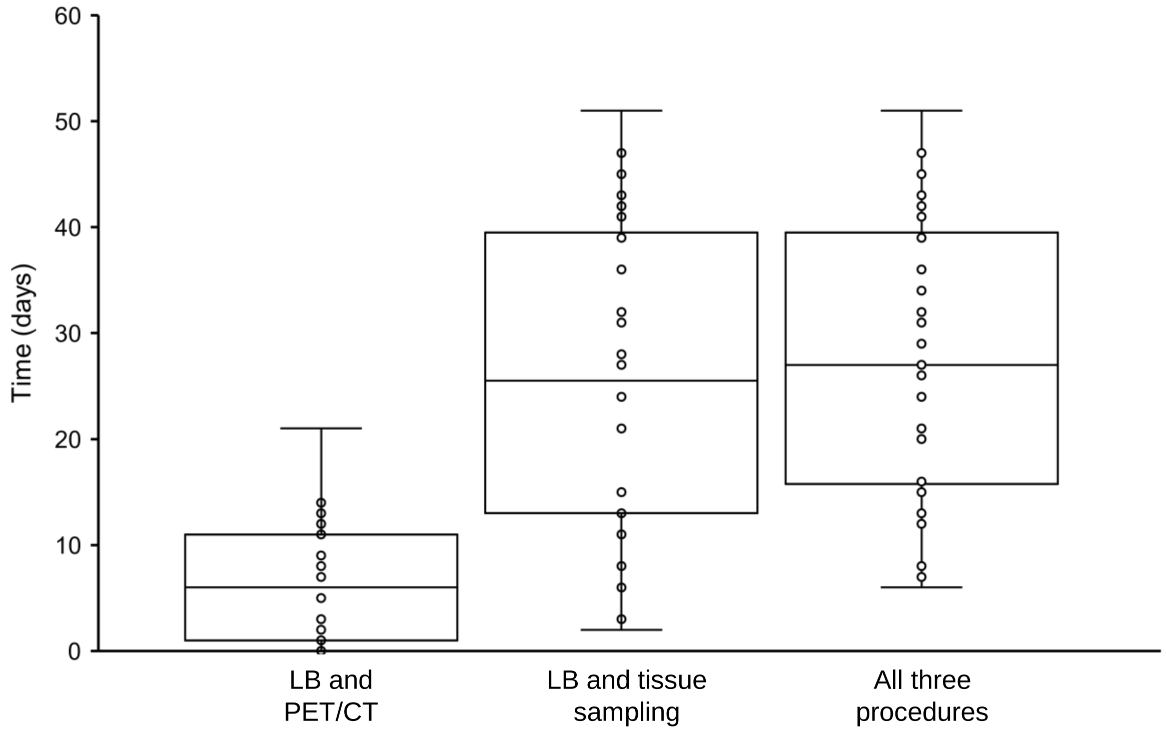

Supplementary Figure S1. Time between liquid biopsy (LB) and PET/CT imaging, LB and tissue sampling and the time for all three study procedures.

Supplementary Table S1. List of detected variants and their variant allele frequencies (VAF) in ctDNA samples.

Gene	Alteration	VAF (%)	Gene	Alteration	VAF (%)
ALK	G1128V	0.87	*PTEN*	loss	-
ATM	E2409*	1.20	*PTEN*	R173C	0.36
ATM	R3008C	0.23	*RAF1*	S259C	0.26
ATM	R337C	2.70	*TERT*	promoter -124C>T	3.70
BRCA1	D693G	48.40	*TERT*	promoter -146C>T	0.32
BRCA2	N257FS*17	48.40	*TP53*	C242R	0.98
CCND1	amplification	NA	*TP53*	D281E	18.70
CDH1	D257G	4.30	*TP53*	E258K	2.00
CDK12	splice site 2610-1G>A	0.29	*TP53*	F212fs*3	1.00
CDKN2A	p14ARF A97V	2.70	*TP53*	G279E	3.70
CDKN2A	p16INK4a H83Y	2.70	*TP53*	H179R	0.18
CDKN2A	p16INK4a W15fs*28	3.40	*TP53*	H193N	4.20
CHEK2	I157T	50.80	*TP53*	L257fs*88,	5.70
CHEK2	splice site 319+1G>C	2.20	*TP53*	N239D	0.36
CHEK2	splice site 792+2T>A	0.99	*TP53*	P316fs*16	2.30
ERBB2	amplification	NA	*TP53*	Q136*	3.90
ERBB2	amplification	NA	*TP53*	R213*	2.80
FGFR2	W290C	24.80	*TP53*	R248W	0.15
FGFR3	R248C	9.70	*TP53*	R249S	5.50
FGFR3	S249C	0.13	*TP53*	R273C	0.66
HRAS	G12C	3.00	*TP53*	R273H	1.30
NF1	P678fs*10	0.44	*TP53*	R273H	0.24
PALB2	splice site 2749 -2A>G	0.15	*TP53*	splice site 673-1G>A	0.91
PIK3CA	D549H	1.00	*TP53*	splice site 783-2A>T	0.19
PIK3CA	E365K	0.29	*TP53*	S127Y	4.70
PIK3CA	E542K	2.20	*TP53*	S241F	7.00
PIK3CA	E545K	0.45	*TP53*	Y205C	0.91
PIK3CA	N1044S	0.12	*TP53*	Y205C	3.50

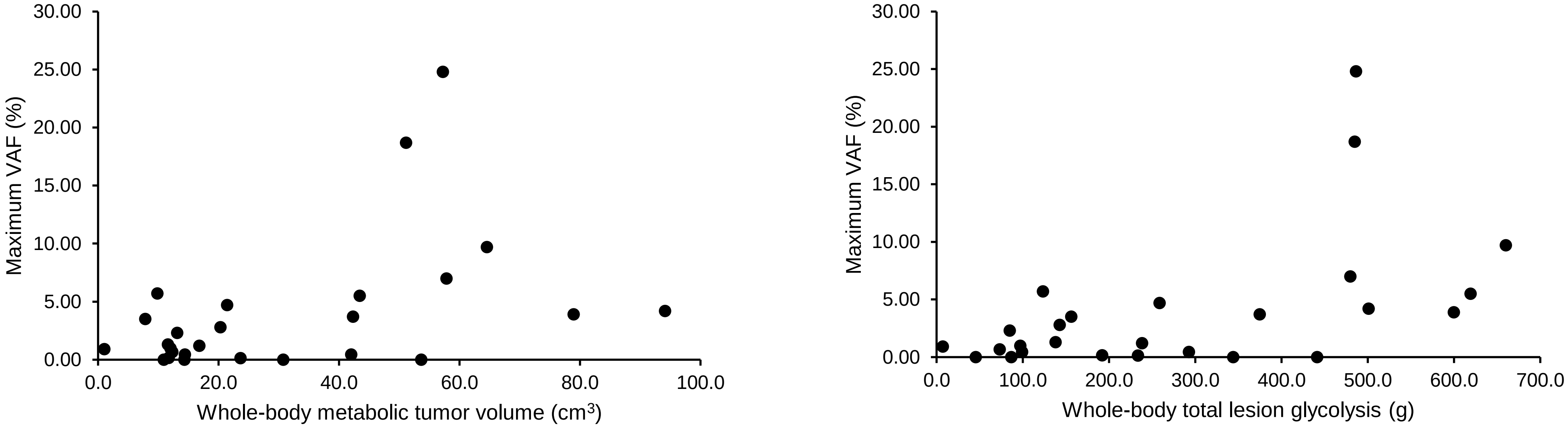

Supplementary Figure S2. The correlations between maximum variant allele frequency (VAF) and whole-body metabolic tumor volume (WB-MTV, r = 0.411 P = 0.037), and maximum VAF and whole-body total lesion glycolysis (WB-TLG, r = 0.549 P = 0.004), excluding the possible germline mutations (see the *Genomic findings in liquid biopsies and tumor samples* in the Results section). The second-highest VAFs are presented from the patients with possible germline mutations.

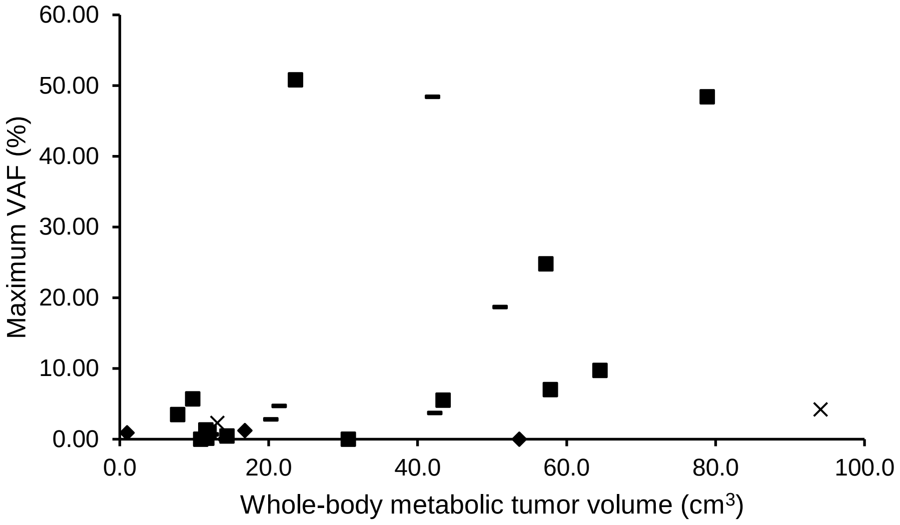

Supplementary Figure S3. The correlations between maximum variant allele frequency (VAF) and whole-body metabolic tumor volume (WB-MTV), and maximum VAF and whole-body total lesion glycolysis (WB-TLG), according to different PET/CT scanners. For scanner A (*square*) and scanner B (*line*), the correlations between maximum VAF and WB-MTV were significant (r = 0.535, P = 0.049 and r = 0.786, P = 0.036, respectively). Similarly, for scanners A and B, the correlations between maximum VAF and WB-TLG were significant (r = 0.601, P = 0.023 and r = 0.786, P = 0.036, respectively). *Diamond* refers to scanner C and *cross* refers to scanner D.

Supplementary Table S2 Median FDG-PET uptake parameters in distinct groups according to liquid biopsy (LB) findings excluding the possible germline mutations.

	**All patients (n=26)**	**Any variant in LB**			**VAF ≥ 1.00% in LB**			**VAF ≥ 5.00% in LB**		
	Median (IQR)	Positive (n=22)	Negative (n=4)	P-value	Positive (n=15)	Negative (n=11)	P-value	Positive (n=6)	Negative (n=20)	P-value
Whole-body										
SUVmax	16.1 (13.3–22.6)	17.2	15.0	0.471	21.1	14.6	0.180	21.8	15.0	0.157
SUVmean	8.4 (6.5–12.0)	8.5	7.2	0.252	8.5	8.0	0.384	9.9	7.8	0.108
MTV	20.9 (11.9–51.7)	20.9	22.5	0.864	42.3	14.3	0.097	54.1	15.6	0.072
TLG	235.8 (98.5–480.9)	235.8	215.1	0.471	374.4	99.0	0.013	485.4	174.0	0.011
Primary tumor										
SUVmax	13.6 (12.2–19.4)	13.6	14.0	0.811	14.0	12.4	0.097	14.3	13.0	0.744
SUVmean	8.4 (6.7–11.7)	8.6	6.3	0.081	9.3	7.2	0.032	9.4	8.0	0.139
MTV	10.5 (5.4–22.1)	9.1	22.5	0.389	10.4	10.6	0.198	15.5	9.1	0.355
TLG	103.6 (38.8–196.1)	103.6	147.5	0.607	138.7	67.3	0.097	137.6	93.5	0.295

VAF, variant allele frequency; SUV, standardized uptake value; MTV, metabolic tumor volume; TLG, total lesion glycolysis

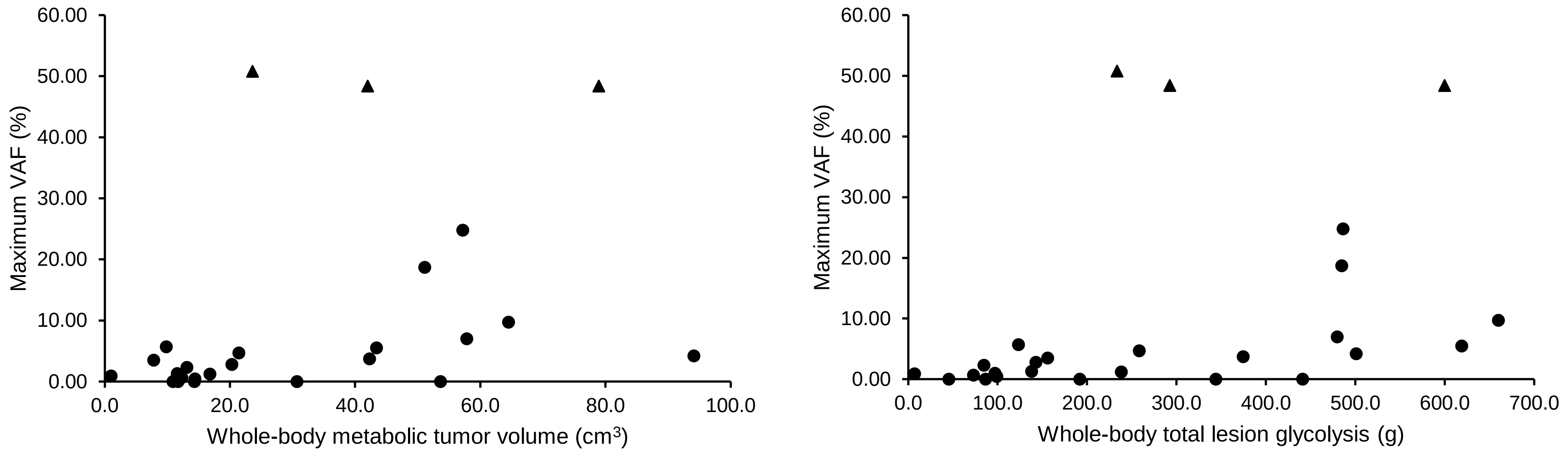

Supplementary Figure S4. The correlations between maximum variant allele frequency (VAF) of concordant variants and whole-body metabolic tumor volume (WB-MTV, r = 0.520 P = 0.006), and maximum VAF of concordant variants and whole-body total lesion glycolysis (WB-TLG, r = 0.583 P = 0.002). The triangels refer to the three patients with possible germline mutations, and the others are labeled with circles.

Supplementary Table S3 Median FDG-PET uptake parameters in distinct groups according to liquid biopsy (LB) findings regarding only concordant variants.

	**All patients (n=26)**	**Any concordant variant in LB**			**Concordant variant with VAF ≥ 1.00% in LB**			**Concordant variant with VAF ≥ 5.00% in LB**		
	Median (IQR)	Positive (n=21)	Negative (n=5)	P-value	Positive (n=16)	Negative (n=10)	P-value	Positive (n=8)	Negative (n=18)	P-value
Whole-body										
SUVmax	16.1 (13.3–22.6)	16.6	15.3	0.900	20.5	14.6	0.165	20.5	14.6	0.144
SUVmean	8.4 (6.5–12.0)	8.5	8.2	0.753	9.0	7.8	0.286	9.7	7.8	0.144
MTV	20.9 (11.9–51.7)	21.4	14.3	0.527	32.9	13.3	0.077	54.1	14.3	0.016
TLG	235.8 (98.5–480.9)	238.2	191.9	0.447	316.4	98.0	0.012	485.4	149.3	0.003
Primary tumor										
SUVmax	13.6 (12.2–19.4)	13.2	15.3	0.569	14.0	12.6	0.182	14.0	13.0	1.000
SUVmean	8.4 (6.7–11.7)	8.5	6.5	0.409	9.0	6.9	0.027	9.0	7.6	0.129
MTV	10.5 (5.4–22.1)	7.8	14.3	0.374	9.1	11.2	0.421	15.5	9.1	0.311
TLG	103.6 (38.8–196.1)	100.5	191.9	0.374	124.3	76.9	0.241	137.6	93.5	0.261

VAF, variant allele frequency; SUV, standardized uptake value; MTV, metabolic tumor volume; TLG, total lesion glycolysis

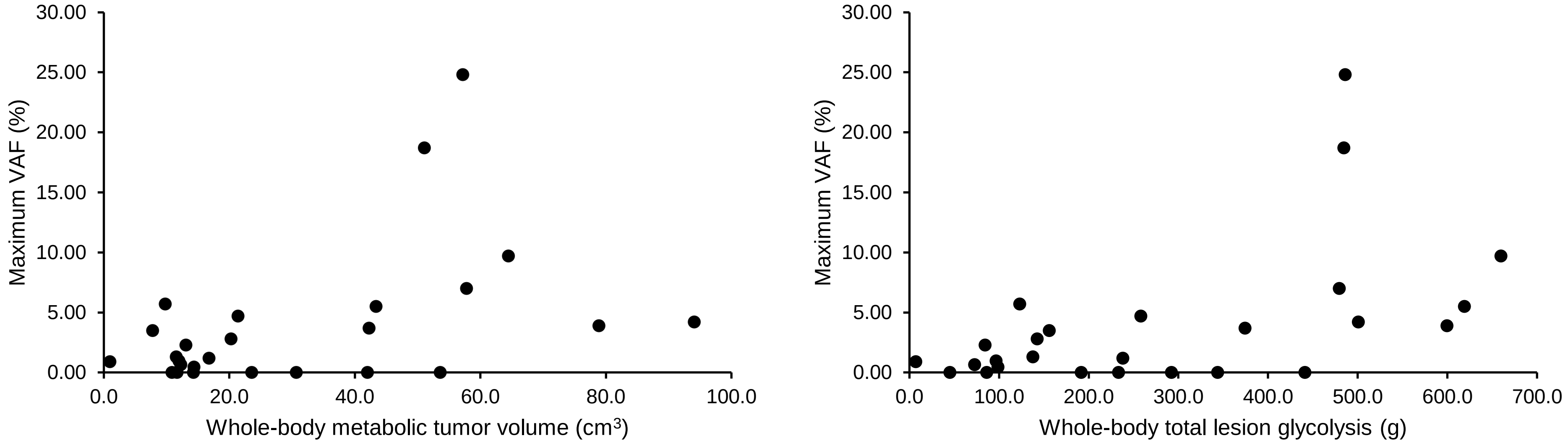

Supplementary Figure S5. The correlations between maximum variant allele frequency (VAF) of concordant variants and whole-body metabolic tumor volume (WB-MTV, r = 0.413 P = 0.036), and maximum VAF of concordant variants and whole-body total lesion glycolysis (WB-TLG, r = 0.539 P = 0.005), excluding the three possible germline mutations. The second-highest VAFs are presented from the patients with possible germline mutations.

Supplementary Table S4 Median FDG-PET uptake parameters in distinct groups according to liquid biopsy (LB) findings regarding only concordant variants and excluding the possible germline mutations.

	**All patients (n=26)**	**Any concordant variant in LB**			**Concordant variant with VAF ≥ 1.00% in LB**			**Concordant variant with VAF ≥ 5.00% in LB**		
	Median (IQR)	Positive (n=20)	Negative (n=6)	P-value	Positive (n=15)	Negative (n=11)	P-value	Positive (n=6)	Negative (n=20)	P-value
Whole-body										
SUVmax	16.1 (13.3–22.6)	16.7	16.0	0.882	21.1	14.6	0.180	21.8	15.0	0.157
SUVmean	8.4 (6.5–12.0)	8.4	9.1	0.929	8.5	8.0	0.384	9.9	7.8	0.108
MTV	20.9 (11.9–51.7)	20.9	18.9	0.614	42.3	14.3	0.097	54.1	15.6	0.072
TLG	235.8 (98.5–480.9)	248.3	212.7	0.457	374.4	99.0	0.013	485.4	174.0	0.011
Primary tumor										
SUVmax	13.6 (12.2–19.4)	13.6	14.0	0.882	14.0	12.4	0.097	14.3	13.0	0.744
SUVmean	8.4 (6.7–11.7)	8.4	7.5	0.457	9.3	7.2	0.032	9.4	8.0	0.139
MTV	10.5 (5.4–22.1)	9.1	13.0	0.790	10.4	10.6	0.198	15.5	9.1	0.355
TLG	103.6 (38.8–196.1)	103.6	139.2	0.790	138.7	67.3	0.097	137.6	93.5	0.295

VAF, variant allele frequency; SUV, standardized uptake value; MTV, metabolic tumor volume; TLG, total lesion glycolysis
